# Thermally Enhanced Darcy-Forchheimer Casson-Water/Glycerine Rotating Nanofluid Flow with Uniform Magnetic Field

**DOI:** 10.3390/mi12060605

**Published:** 2021-05-23

**Authors:** Anum Shafiq, Ghulam Rasool, Hammad Alotaibi, Hassan M. Aljohani, Abderrahim Wakif, Ilyas Khan, Shakeel Akram

**Affiliations:** 1 School of Mathematics and Statistics, Nanjing University of Information Science and Technology, Nanjing 210044, China; anumshafiq@ymail.com; 2 Binjiang College, Nanjing University of Information Science and Technology, Wuxi 214105, China; 3 Department of Mathematics, College of Science, Taif University, P.O. Box 11099, Taif 21944, Saudi Arabia; hmjohani@tu.edu.sa; 4 Laboratory of Mechanics, Faculty of Sciences Aïn Chock, Hassan II University, Mâarif, B.P. 5366 Casablanca, Morocco; wakif.abderrahim@gmail.com; 5 Department of Mathematics, College of Science Al-Zulfi, Majmaah University, Al-Majmaah 11952, Saudi Arabia; 6 College of Electrical Engineering, Sichuan University, Chengdu 610065, China; shakeel.akram@scu.edu.cn

**Keywords:** Darcy-Forchheimer theory, carbon nanotubes, nanofluid, magnetohydrodynamics, thermal radiation

## Abstract

This numerical study aims to interpret the impact of non-linear thermal radiation on magnetohydrodynamic (MHD) Darcy-Forchheimer Casson-Water/Glycerine nanofluid flow due to a rotating disk. Both the single walled, as well as multi walled, Carbon nanotubes (CNT) are invoked. The nanomaterial, thus formulated, is assumed to be more conductive as compared to the simple fluid. The properties of effective carbon nanotubes are specified to tackle the onward governing equations. The boundary layer formulations are considered. The base fluid is assumed to be non-Newtonian. The numerical analysis is carried out by invoking the numerical Runge Kutta 45 (RK45) method based on the shooting technique. The outcomes have been plotted graphically for the three major profiles, namely, the radial velocity profile, the tangential velocity profile, and temperature profile. For skin friction and Nusselt number, the numerical data are plotted graphically. Major outcomes indicate that the enhanced Forchheimer number results in a decline in radial velocity. Higher the porosity parameter, the stronger the resistance offered by the medium to the fluid flow and consequent result is seen as a decline in velocity. The Forchheimer number, permeability parameter, and porosity parameter decrease the tangential velocity field. The convective boundary results in enhancement of temperature facing the disk surface as compared to the ambient part. Skin-friction for larger values of Forchheimer number is found to be increasing. Sufficient literature is provided in the introduction part of the manuscript to justify the novelty of the present work. The research greatly impacts in industrial applications of the nanofluids, especially in geophysical and geothermal systems, storage devices, aerospace engineering, and many others.

## 1. Introduction

The contribution of nanomaterials (Nanofluids) in industry and engineering is very diversified. A lot of advantages have been noted by induction of nanomaterials in fluid flow analysis. Pioneered by Choi [1], the term nanofluid is also named as the nanomaterials subject to the type of nanoparticles that are used in the formulation procedure. The chore and basic property of nanomaterials is very important, i.e., enhanced thermal conductivity. The base fluids such as water, ethylene, toluene, and kerosene oil are the most commonly used base fluids in this regard and there are several related works that have been reported in the recent past to practically implement the idea of nanofluids and nanomaterials. For instance, some important studies are mentioned in the following lines. Lin et al. [2] reported discussion on the results obtained for MHD Transient Pseudo-Plastic nanofluid flow giving a highlight of the heat transfer properties and the impact of drag force in this transport. Bai et al. [3] analyzed the Brownian diffusion and the thermophoresis in radiative MHD Maxwell type nanofluid flow. They highlighted both the heat and mass transport phenomena in fluid flow procedures and provided sufficient numerical data to adhere the physical quantities. Madhu et al. [4] analyzed the non-Newtonian fluid behavior using the Maxwell model. The important aspect of this study is the consequence of MHD, as well as thermal radiation, on the flow attributes, especially the drag force component. Sheikholeslami et al. [5] reported the impact of MHD and radiation on Darcy-type flow of nanomaterials using controlled volume based finite element method (CVFEM) scheme. This scheme is more accurate as compared to the conventional analytic methods. Thus, the results were more precise and accurate for implementation in respective industrial applications. Williamson nanofluid flow using bi-directional stretching surface using the Brownian diffusion and thermophoresis are the main features of study reported by Hayat et al. [6] where they discussed three dimensional fluid flow analysis. The three dimensional nanofluid convection in natural flow has been reported by Zadi et al. [7]. The second important aspect of this study is related with studies carried out using disks and, here, this disk is assumed to be permeable under the definition of Darcian medium. Such formulations are highly important in geophysical and geothermal systems, storage devices, aerospace engineering, crystal growing procedures, medical instruments, and many food processing techniques that are based on porous mediums. The purpose behind such formulations is heat and mass transfer analysis by rotating frame. Several studies are available in literature on such formulations. Turkyilmazoglu and Senel [8] reported significant study on viscous nanofluid flow bounded by a porous disk using the usual Van Karman type of transformations. Bödewadt et al. [9] reported boundary layer fluid flow analysis using the rotating frame/disk. This study was used as a base study for investigation of fluid flow analysis using stationary disk by Mustafa et al. [10]. The use of partial slip conditions in nanofluid flow bounded by rotating disk under the impact of MHD is an important study reported by Mustafa [11]. Dogonchi et al. [12] interrogated the impact of heat convection using the magnetic field effect and shape and size factor of the used nanoparticles, using a cavity as the core surface. The importance of Casson material can not be neglected in this research, which is assumed to be a major factor for fluid flow analysis under the present formulation. It has several important applications in industry, such as metallurgy, food processing, bio-engineering and drilling operations, etc. The mixing of Casson material with Water-Glycerine, etc., is the base of Casson nanofluid. This so formulated nanomaterial has shear thinning properties, having infinite values of viscosity even at a zero shearing rate and deformation, below to which there is no flow. Besides, the importance of a porous (Darcy) medium is yet another very important phenomena in fluid flow analysis. It has received utmost attention in the last few decades. The efficiency of the typical energy systems is, therefore, enhanced using such formulations. Law of Darcy is valid for very small Reynolds number and, therefore, the high speed flows cannot be dealt under such theories. Therefore, the improvement was genuinely required which was dealt by Forchheimer [13] for high flow rates. Therefore, the combined Darcy-Forchheimer relation is way more effective to deal with fluid flow analysis in porous medium for moderate flow rates. Several studies have been reported on the significance of Darcy-Forchheimer medium, such as the impact of Cattaneo-Christov model in fluid flow analysis in Darcy-Medium has been reported by Shehzad et al. [14]. Bakar et al. [15] reported boundary layer approximation in stagnation point flow under Darcy-Forchheimer model of heat and mass flux. Hayat et al. [16] reported the significance of Cattaneo-Christov model in Darcy-Forchheimer model using variable thermal conductivity. In their study, Chamkha et al. [17] categorically reported the impact of radiation on the nanofluid flow via wedge using a Darcy type medium. In another article, Chamkha et al. [18] discussed the significance of thermal radiation in the mixed convective nanofluid flow having porous medium. The concept on CNTs was first revealed in early 1991 leading to extensive investigations for its not known properties. The micro-level structure of CNT is usually seen in cylindrical shape rolled from the single sheet called graphite. CNTs are usually divided into two categories as single wall nanotubes and multi walled nanotubes. For sure, the multi wall structure is more complicated as compared to single wall nanotubes. In theoretical fluid mechanics, the nanotubes are analyzed by their properties pre-defined for a particular problem. Several studies related to the structure and the applications of these tubes are available in literature. One can read [19,20,21,22,23,24,25,26,27,28,29,30,31,32,33,34,35] and cross reference therein.

Up till now, the literature survey indicates that there is a gap of study in the context of heat and mass transfer investigation on the Casson-Water/Glycerine nanofluids convection due to radially stretching disk. Therefore, the objective of present investigation is clear and novel, i.e., to explore the variation imparted by Carbon nanotubes, non-linear thermal radiation and Darcy-Forchheimer relation on Casson-Water/Glycerine nanofluid flow due to radially stretching disk. These formulations are highly important in geophysical and geothermal systems, storage devices, aerospace engineering, crystal growing procedures, medical instruments, and many food processing techniques that are based on a porous medium to help understand the fluid flow, heat transfer, especially the drag force intensity at the surface, which is in contact with the fluid. Numerical scheme is implemented for finding the solutions of so-formulated problems. The analysis is carried out via graphical display of the results for various parameters and their impact on the three profile of nanofluids in boundary layer approximations. Furthermore, the variation in skin-friction and Nusselt number is noted via graphical display. The article concludes with physical justifications and major findings of the study.

## 2. Problem Formulation

**Figure 1:**
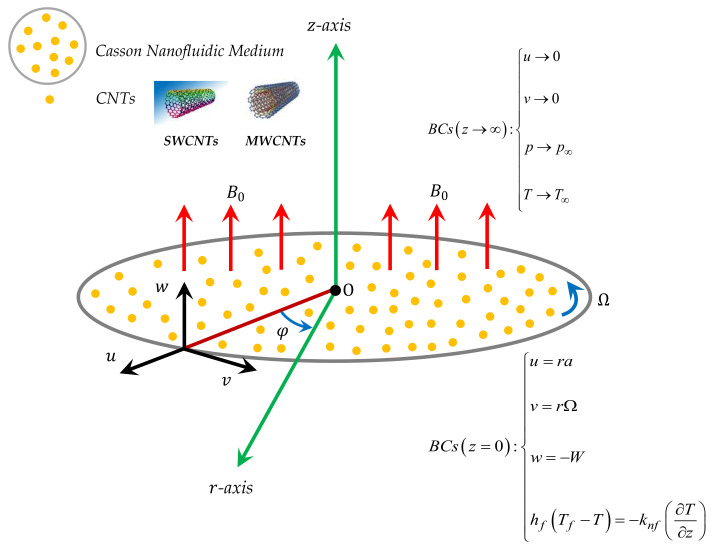
Physical model and coordinate system.

In this numerical investigation, we include the influence of non-linear thermal radiation and Carbon nanotubes on viscous incompressible Darcy-Forchheimer nanofluid flow bounded by rotating disk. Thermal convection is analyzed and convective boundary is invoked. The porosity factor appears highly under the implementation of Darcy-Forchheimer model. The formulation is based on two type of materials, i.e., water and glycerine, respectively. The single- and multi-walled carbon nanotubes are considered whose properties are given in the Table 1. The velocity components are taken as (u, v, w), in the direction of (r,ϕ,z), respectively. The rotation of disk is assumed at z=0. One can see the physical scenario in Figure 1.

Consider,

(1) $$\frac{\partial u}{\partial r}+\frac{u}{r}+\frac{\partial w}{\partial z}=0,$$

(2) $$\begin{array}{cc} u\frac{\partial u}{\partial r}-\frac{v^{2}}{r}+w\frac{\partial u}{\partial z} & =\frac{-1}{\rho_{nf}}\frac{\partial p}{\partial r}+\left(1+\frac{1}{\beta_{1}}\right)\frac{\mu_{nf}}{\rho_{nf}}\left(\frac{\partial^{2}u}{\partial r^{2}}+\frac{1}{r}\frac{\partial u}{\partial r}-\frac{u}{r^{2}}+\frac{\partial^{2}u}{\partial z^{2}}\right)\\ \; & +\frac{{\left(\rho \beta \right)}_{nf}}{\rho_{nf}}g_{1}\left(T-T_{\infty }\right)-\frac{\mu_{nf}}{\rho_{nf}}\frac{u}{K^{*}}-F^{*}u^{2}-\frac{\sigma B_{0}^{2}}{\rho }u, \end{array}$$

(3) $$\begin{array}{cc} u\frac{\partial v}{\partial r}+\frac{uv}{r}+w\frac{\partial v}{\partial z} & =\left(1+\frac{1}{\beta_{1}}\right)\frac{\mu_{nf}}{\rho_{nf}}\left(\frac{\partial^{2}v}{\partial r^{2}}+\frac{1}{r}\frac{\partial v}{\partial r}-\frac{v}{r^{2}}+\frac{\partial^{2}v}{\partial z^{2}}\right)\\ \; & +\frac{{\left(\rho \beta \right)}_{nf}}{\rho_{nf}}g_{1}\left(T-T_{\infty }\right)-\frac{\mu_{nf}}{\rho_{nf}}\frac{v}{K^{*}}-F^{*}v^{2}-\frac{\sigma B_{0}^{2}}{\rho }v, \end{array}$$

(4) $$\begin{array}{c} u\frac{\partial w}{\partial r}+w\frac{\partial w}{\partial z}=-\frac{1}{\rho_{nf}}\frac{\partial p}{\partial z}+\left(1+\frac{1}{\beta_{1}}\right)\frac{\mu_{nf}}{\rho_{nf}}\left(\frac{\partial^{2}w}{\partial r^{2}}+\frac{1}{r}\frac{\partial w}{\partial r}+\frac{\partial^{2}w}{\partial z^{2}}\right), \end{array}$$

(5) $$\begin{array}{cc} {\left(\rho C_{p}\right)}_{nf}\left(u\frac{\partial T}{\partial r}+w\frac{\partial T}{\partial z}\right) & =\left(k_{nf}+\frac{16\sigma^{*}T^{3}}{3k^{*}}\right)\left(\frac{\partial^{2}T}{\partial r^{2}}+\frac{1}{r}\frac{\partial T}{\partial r}+\frac{\partial^{2}T}{\partial z^{2}}\right)\\ \; & +\frac{16\sigma^{*}T^{2}}{k^{*}}\left[{\left(\frac{\partial T}{\partial z}\right)}^{2}+{\left(\frac{\partial T}{\partial r}\right)}^{2}\right]+\left(1+\frac{1}{\beta_{1}}\right)2\mu_{nf}\left[{\left(\frac{\partial u}{\partial r}\right)}^{2}+{\left(\frac{\partial w}{\partial z}\right)}^{2}+\frac{u^{2}}{r^{2}}\right]\\ \; & +\mu_{nf}\left(1+\frac{1}{\beta_{1}}\right)\left[{\left(\frac{\partial v}{\partial z}\right)}^{2}+{\left(\frac{\partial w}{\partial r}+\frac{\partial u}{\partial z}\right)}^{2}+{\left[r\frac{\partial }{\partial r}\left(\frac{v}{r}\right)\right]}^{2}\right]. \end{array}$$

The boundary conditions are,

(6) $$v=r\Omega ,\;\;u=ra,\;\;w=-W,\;\;h_{f}\left(T_{f}-T\right)=-k_{nf}\left(\frac{\partial T}{\partial z}\right),\;\;\mathrm{at}\;z=0.$$

(7) $$u\to 0,\;\;v\to 0,\;\;p\to p_{\infty },\;\;T\to T_{\infty },\;\;\mathrm{as}\;\;z\to \infty .$$

The effective Carbon nanotubes are (see for reference Shaw et al. [36]),

(8) $$\begin{array}{cc} \mu_{nf} & =\frac{\mu_{f}}{{\left(1-\phi \right)}^{2.5}},\\ \rho_{nf} & ={\left(1-\phi \right)}_{nf}+\phi \rho_{CNT},\\ \alpha_{nf} & =\frac{k_{nf}}{\rho_{nf}}{\left(C_{p}\right)}_{nf},\\ \nu_{nf} & =\frac{\mu_{nf}}{\rho_{nf}},\\ \frac{K_{nf}}{K_{f}} & =\frac{\left(1-\phi \right)+2\phi \frac{K_{CNT}}{K_{CNT}-K_{f}}\log\frac{K_{CNT}+K_{f}}{2K_{f}}}{\left(1-\phi \right)+2\phi \frac{K_{f}}{K_{CNT}-K_{f}}\log\frac{K_{CNT}+K_{f}}{2K_{f}}},\\ {\left(\rho \beta \right)}_{nf} & =\left(1-\phi \right){\left(\rho \beta \right)}_{f}+\phi {\left(\rho \beta \right)}_{S_{1}},\\ {\left(\rho C_{p}\right)}_{nf} & =\left(1-\phi \right){\left(\rho C_{p}\right)}_{f}+\phi {\left(\rho C_{p}\right)}_{CNT}, \end{array}$$

Using the following transformations,

(9) $$\begin{array}{cc} u & =r\Omega f^{\prime },\;\;v=r\Omega g,\;\;w=-\sqrt{2\Omega \nu_{f}}f,\\ \; & p=p_{\infty }-\mu_{f}\Omega P,\;\;T=T_{\infty }-\left(T_{\infty }-T_{f}\right)\theta ,\\ \; & \eta =\sqrt{\frac{2\Omega }{\nu_{f}}}z. \end{array}$$

the final non-dimensional equations are,

(10) $$\begin{array}{cc} \; & \frac{1}{{\left(1-\phi \right)}^{2.5}\left(1-\phi +\phi \left(\frac{\rho_{CNT}}{\rho_{f}}\right)\right)}\left(1+\frac{1}{\beta_{1}}\right)\left(2f^{\prime \prime \prime }-Kf^{\prime }\right)+2ff^{\prime \prime }-{f^{\prime }}^{2}\\ \; & +g^{2}+\frac{\left(1-\phi +\frac{{\left(\rho \beta \right)}_{CNT}\phi }{{\left(\rho \beta \right)}_{f}}\right)}{1-\phi +\frac{\rho_{CNT}}{\rho_{f}}\phi }\lambda \theta -F_{r}{f^{\prime }}^{2}-\frac{M_{1}^{2}}{1-\phi +\phi \frac{\rho_{CNT}}{\rho_{f}}}f=0, \end{array}$$

(11) $$\begin{array}{cc} \; & \frac{1}{{\left(1-\phi \right)}^{2.5}\left(1-\phi +\phi \left(\frac{\rho_{CNT}}{\rho_{f}}\right)\right)}\left(1+\frac{1}{\beta_{1}}\right)\left(g^{\prime \prime }-Kg\right)+2fg^{\prime }-2f^{\prime }g+\frac{\left(1-\phi +\phi \frac{{\left(\rho \beta \right)}_{CNT}\phi }{{\left(\rho \beta \right)}_{f}}\right)}{1-\phi +\frac{\rho_{CNT}\phi }{\rho_{f}}\phi }\lambda \theta \\ \; & -F_{r}g^{2}-\frac{M_{1}^{2}}{1-\phi +\phi \frac{\rho_{CNT}}{\rho_{f}}}g=0, \end{array}$$

(12) $$\begin{array}{cc} \; & \frac{\frac{K_{nf}}{K_{f}}}{\left(1-\phi +\phi \left(\frac{{\left(\rho C_{p}\right)}_{CNT}}{{\left(\rho C_{p}\right)}_{f}}\right)\right)}\left(1+\frac{4}{3}\frac{K_{f}}{K_{nf}}R_{1}{\left[1+\left(\theta_{f}-1\right)\theta \right]}^{3}\right)\theta^{\prime \prime }\\ \; & +\frac{4R_{1}}{1-\phi +\phi \frac{{\left(\rho C_{p}\right)}_{CNT}}{{\left(\rho C_{p}\right)}_{f}}}\left[{\left(1+\left(\theta_{f}-1\right)\theta \right)}^{2}\left(\theta_{f}-1\right){\left(\theta^{\prime }\right)}^{2}\right]\\ \; & +Pr\left[f\theta^{\prime }+\frac{6}{{\left(1-\phi \right)}^{2.5}}\left(1+\frac{1}{\beta_{1}}\right)\frac{Ec}{Re}{\left(f^{\prime }\right)}^{2}+2\left(1+\frac{1}{\beta_{1}}\right)Ec\frac{1}{{\left(1-\phi \right)}^{2.5}}\left[{\left(f^{\prime \prime }\right)}^{2}+{g^{\prime }}^{2}\right]\right]=0. \end{array}$$

Such that,

(13) $$g=1,\;\;f^{\prime }=\delta_{1},\;\;f=\delta_{1},\;\;-B_{i}\left(1-\theta \right)=\frac{K_{nf}}{K_{f}}\theta^{\prime }\;\;\mathrm{at}\;\eta =0,$$

(14) $$g\to 0,\;\;f^{\prime }\to 0,\;\;\theta \to 0,\;\;\mathrm{as}\;\eta \to \infty .$$

where,

(15) $$\begin{array}{cc} F_{r} & =\frac{Cd}{\sqrt{K^{*}}},\;\;Pr=\frac{\nu_{f}}{\alpha_{f}},\;\;R_{1}=\frac{4\sigma^{*}T_{\infty }^{3}}{K^{*}K_{f}},\\ \; & S_{1}=\frac{W}{\sqrt{2\Omega \nu_{f}}},\;\;Ec=\frac{r^{2}\Omega^{2}}{{\left(C_{p}\right)}_{f}T_{\infty }\left(\theta_{f}-1\right)},\;\;\delta_{1}=\frac{a}{\Omega },\\ \; & Bi=\frac{h_{f}}{K_{f}}\sqrt{\frac{\nu_{f}}{2\Omega }}, \end{array}$$

are Forchheimer number, Prandtl number, nonlinear radiation factor, Suction parameter, Eckert number, stretching strength parameter, and Biot number, respectively. The physical quantities are,

(16) $$Re_{r}^{1/2}C_{f}=\frac{1}{{\left(1-\phi \right)}^{2.5}}\left(1+\frac{1}{\beta }\right)\sqrt{{\left(G^{\prime }\left(0\right)\right)}^{2}+{\left(f^{\prime \prime }\left(0\right)\right)}^{2}},$$

(17) $$Re_{r}^{-1/2}Nu_{r}=-\left[\frac{K_{nf}}{K_{f}}+\frac{4}{3}R_{1}{\left[1+\left(\theta_{f}-1\right)\theta \left(0\right)\right]}^{3}\right]\theta^{\prime }\left(0\right).$$

## 3. Solution Methodology

The numerical RK45 scheme, together with the shooting technique, is implemented for final solutions of the problems. In order to gain a clear physical insight, firstly, the above Equations (11) and (12), along with the boundary conditions (13) and (14), are converted to an initial value problem and then solved numerically by means of the fourth-order Runge–Kutta method coupled with the shooting technique, with a systematic estimate of $f^{\prime \prime }\left(0\right)$ and θ(0) according to the corresponding boundary conditions at $f^{\prime }\left(\infty \right)$ and θ(∞) with the Newton–Raphson shooting technique. In this method, it is necessary to choose a suitable finite value for η→∞, say $\eta_{\infty }$. If the boundary conditions at infinity are not satisfied, then the numerical routine uses the Newton–Raphson method to calculate the corrections to the estimated values of $f^{\prime \prime }\left(0\right)$ and θ(0). This process is repeated iteratively until convergence is achieved to a specified accuracy, with order 10–5. Assuming the governing parameters are as follows,

(18) $$j_{1}=f,j_{2}=f^{\prime },j_{3}=f^{\prime \prime },j_{4}=g,j_{5}=g^{\prime },j_{6}=\theta ,j_{7}=\theta^{\prime }$$

then, higher order of governing equations can be written as follows:

(19) $$\begin{array}{cc} f^{\prime \prime \prime } & =-\frac{1}{2\frac{1}{{\left(1-\phi \right)}^{2.5}\left(1-\phi +\phi \left(\frac{\rho_{CNT}}{\rho_{f}}\right)\right)}\left(1+\frac{1}{\beta_{1}}\right)}\\ \; & \left[-K\left(1+\frac{1}{\beta_{1}}\right)\frac{1}{{\left(1-\phi \right)}^{2.5}\left(1-\phi +\phi \left(\frac{\rho_{CNT}}{\rho_{f}}\right)\right)}j_{2}+2j_{1}j_{3}-j_{1}^{2}+j_{4}^{2}\right]\\ \; & -\frac{1}{2\frac{1}{{\left(1-\phi \right)}^{2.5}\left(1-\phi +\phi \left(\frac{\rho_{CNT}}{\rho_{f}}\right)\right)}\left(1+\frac{1}{\beta_{1}}\right)}\left[\frac{\left(1-\phi +\frac{{\left(\rho \beta \right)}_{CNT}\phi }{{\left(\rho \beta \right)}_{f}}\right)}{1-\phi +\frac{\rho_{CNT}\phi }{\rho_{f}}}\lambda j_{6}-Frj_{2}^{2}-\frac{M_{1}^{2}}{1-\phi +\phi \frac{\rho_{CNT}}{\rho_{f}}}j_{1}\right], \end{array}$$

(20) $$\begin{array}{cc} g^{\prime \prime } & =-\frac{1}{\frac{1}{{\left(1-\phi \right)}^{2.5}\left(1-\phi +\phi \left(\frac{\rho_{CNT}}{\rho_{f}}\right)\right)}\left(1+\frac{1}{\beta_{1}}\right)}\\ \; & \left[-K\left(1+\frac{1}{\beta_{1}}\right)\frac{1}{{\left(1-\phi \right)}^{2.5}\left(1-\phi +\phi \left(\frac{\rho_{CNT}}{\rho_{f}}\right)\right)}j_{4}+2j_{1}j_{5}-2j_{2}j_{4}\right]\\ \; & -\frac{1}{\frac{1}{{\left(1-\phi \right)}^{2.5}\left(1-\phi +\phi \left(\frac{\rho_{CNT}}{\rho_{f}}\right)\right)}\left(1+\frac{1}{\beta_{1}}\right)}\left[\frac{\left(1-\phi +\frac{{\left(\rho \beta \right)}_{CNT}\phi }{{\left(\rho \beta \right)}_{f}}\right)}{1-\phi +\frac{\rho_{CNT}\phi }{\rho_{f}}}\lambda j_{6}-Frj_{4}^{2}-\frac{M_{1}^{2}}{1-\phi +\phi \frac{\rho_{CNT}}{\rho_{f}}}j_{4}\right], \end{array}$$

(21) $$\begin{array}{cc} \theta^{\prime \prime } & =-\frac{Pr}{\frac{\frac{K_{nf}}{K_{f}}}{\left(1-\phi +\phi \left(\frac{{\left(\rho C_{p}\right)}_{CNT}}{{\left(\rho C_{p}\right)}_{f}}\right)\right)}\left(1+\frac{4}{3}\frac{K_{f}}{K_{nf}}R_{1}{\left[1+\left(\theta_{f}-1\right)j_{6}\right]}^{3}\right)}\left(\frac{4R_{1}}{1-\phi +\phi \frac{{\left(\rho C_{p}\right)}_{CNT}}{{\left(\rho C_{p}\right)}_{f}}}{\left(1+\left(\theta_{f}-1\right)j_{6}\right)}^{2}j_{7}^{2}\right)\\ \; & \frac{-Pr}{\frac{\frac{K_{nf}}{K_{f}}}{\left(1-\phi +\phi \left(\frac{{\left(\rho C_{p}\right)}_{CNT}}{{\left(\rho C_{p}\right)}_{f}}\right)\right)}\left(1+\frac{4}{3}\frac{K_{f}}{K_{nf}}R_{1}{\left[1+\left(\theta_{f}-1\right)j_{6}\right]}^{3}\right)}\left(j_{1}j_{7}+\frac{6}{{\left(1-\phi \right)}^{2.5}}\left(1+\frac{1}{\beta_{1}}\right)\frac{Ec}{Re}j_{2}^{2}\right)\\ \; & \frac{-Pr}{\frac{\frac{K_{nf}}{K_{f}}}{\left(1-\phi +\phi \left(\frac{{\left(\rho C_{p}\right)}_{CNT}}{{\left(\rho C_{p}\right)}_{f}}\right)\right)}\left(1+\frac{4}{3}\frac{K_{f}}{K_{nf}}R_{1}{\left[1+\left(\theta_{f}-1\right)j_{6}\right]}^{3}\right)}\left(2\left(1+\frac{1}{\beta_{1}}\right)Ec\frac{1}{{\left(1-\phi \right)}^{2.5}}\left(j_{3}^{2}+j_{5}^{2}\right)\right) \end{array}$$

Thus, the above mentioned three equations are used to write down the system of non linear governing ODEs in the form of a matrix subject to the converted boundary conditions according to the new parameters, and solved by using the numerical scheme. The skin friction coefficient and the Nusselt number are also converted accordingly.

## 4. Results and Discussion

Here in, Casson-water/glycerine MHD Darcy-Forchheimer nanofluid flow analysis subject to a rotating frame is considered. The rate of heat transfer and skin-friction are analyzed. The graphical display of results gives the impact of various parameters involved in the flow model on the main profiles of momentum and energy. The numerical RK45 scheme is invoked to obtain the requisite solutions of the governing non-linear ordinary differential equations. The graphs are sketched from the final solutions to analyze the impact of various parameters on fluid flow profiles. It is pertinent to note that solid lines represent the carbon nanotubes—water dilution, while the dashed lines are used for carbon nanotubes—Glycerine dilute, respectively.

### 4.1. Radial Velocity

**Figure 2:**
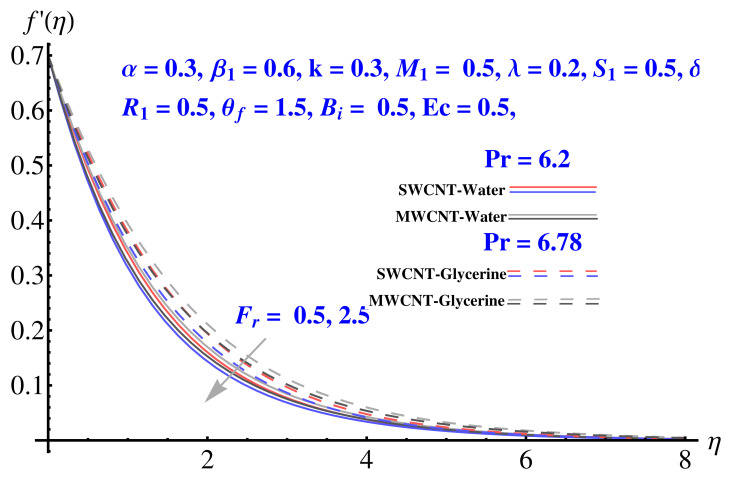
Impact of Fr on radial velocity.

**Figure 3:**
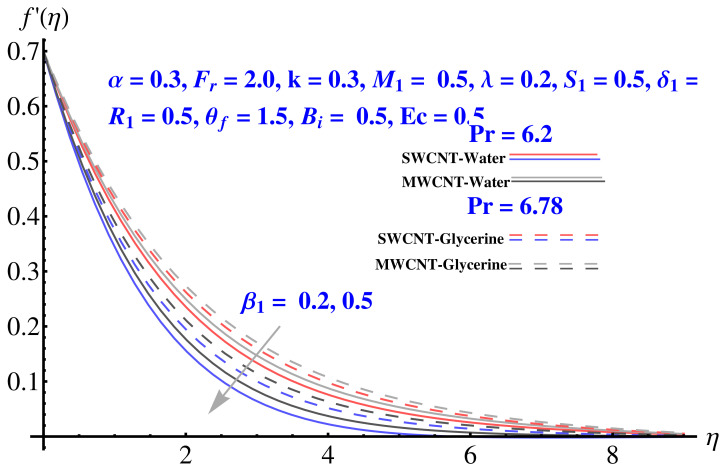
Impact of $\beta_{1}$ on radial velocity.

**Figure 4:**
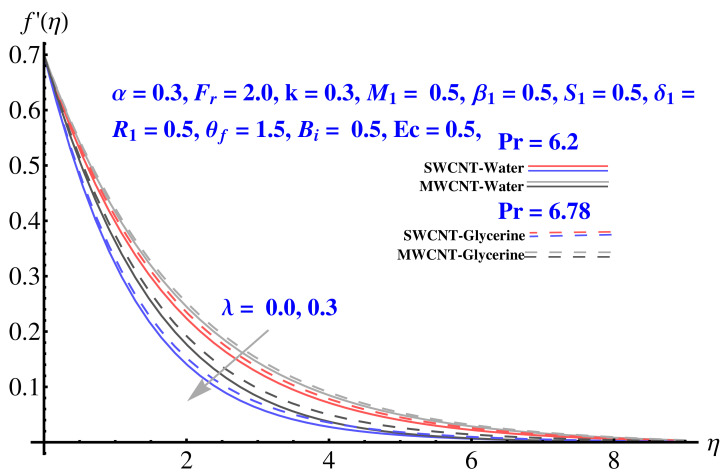
Impact of λ on radial velocity.

**Figure 5:**
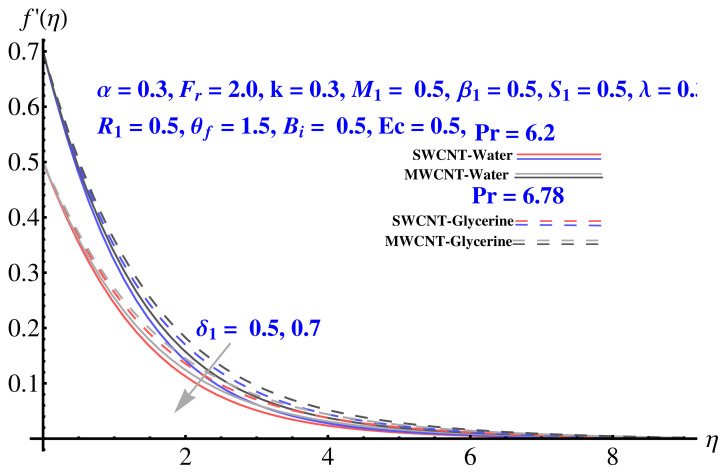
Impact of $\delta_{1}$ on radial velocity.

**Figure 6:**
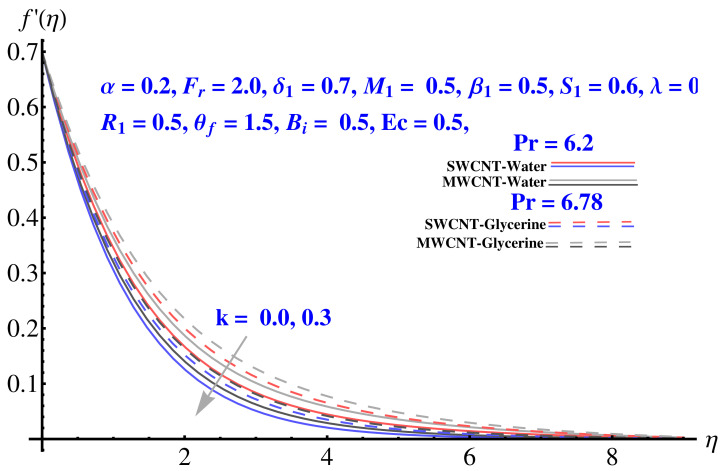
Impact of *k* on radial velocity.

In particular, Figure 2 gives the impact of the Forchheimer number on the momentum boundary layer in the context of radial velocity field. The enhanced Forcheimer number physically relates with more frictional force offered to the fluid in the opposite direction of the movement. Clearly, a decline in both cases, i.e., SWCNTs and MWCNTs can be seen in the figure. Figure 3 represents the behavior of velocity profile subject to variation in Casson parameter. Both, the solid and dashed lines present a declining trend. Physically, the elevated Casson parameter means a reduction in yield stress which in turns correspond to a Newtonian fluid, consequently, the fluid velocity undergoes a restriction. Figure 4 gives the variation in velocity field subject to augmented values of porosity factor. The larger the porosity parameter, the larger the resistance offered by the medium to the fluid flow and consequent result is decline in velocity. Both the cases behave in similar trends. The impact of stretching strength parameter on radial velocity is given in Figure 5. The stronger stretching rate corresponds to declination in the radial component of velocity. Away from disk, the result is significant decline in velocity profile. Figure 6 corresponds to the significance of permeability parameter (K) in the radial velocity field. The velocity profile shows drastic declination in both cases when the values of K are increased. Larger values of K correspond to the dense porous matrix, which in turn offers intensive resistance to the fluid flow, and consequently a stronger retardation is faced by the fluid movement.

### 4.2. Tangential Velocity

**Figure 7:**
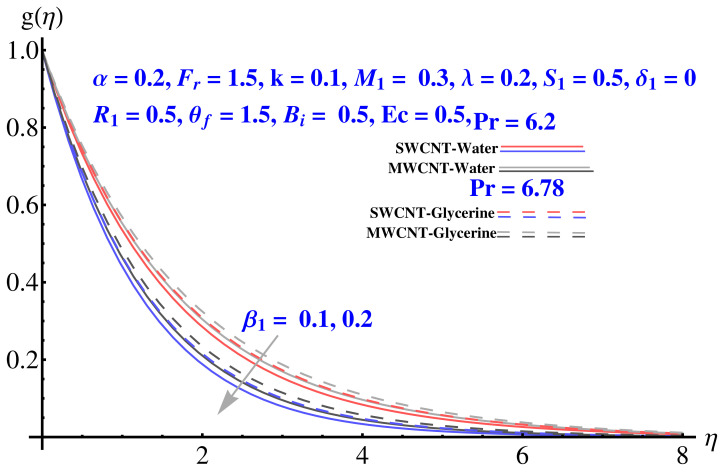
Impact of $\beta_{1}$ on tangential velocity.

**Figure 8:**
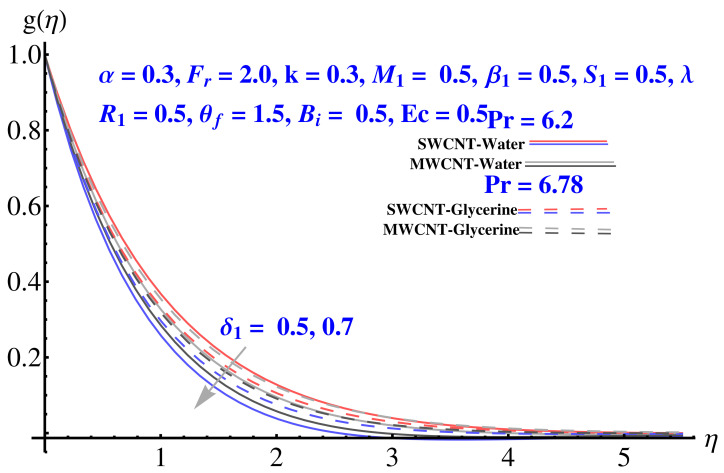
Impact of $\delta_{1}$ on tangential velocity.

**Figure 9:**
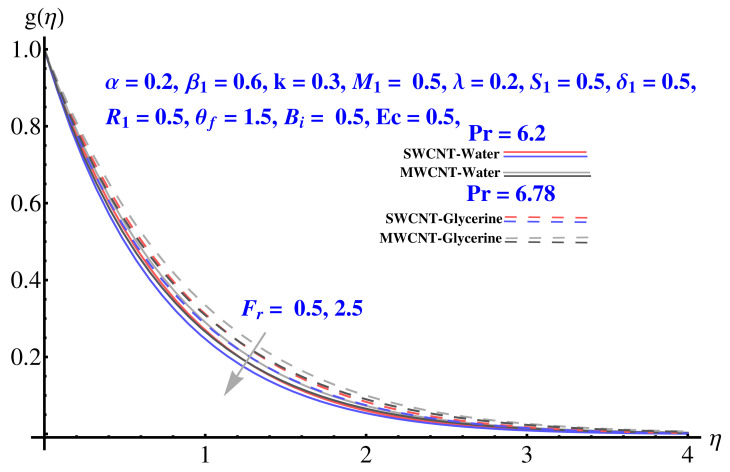
Impact of Fr on tangential velocity.

**Figure 10:**
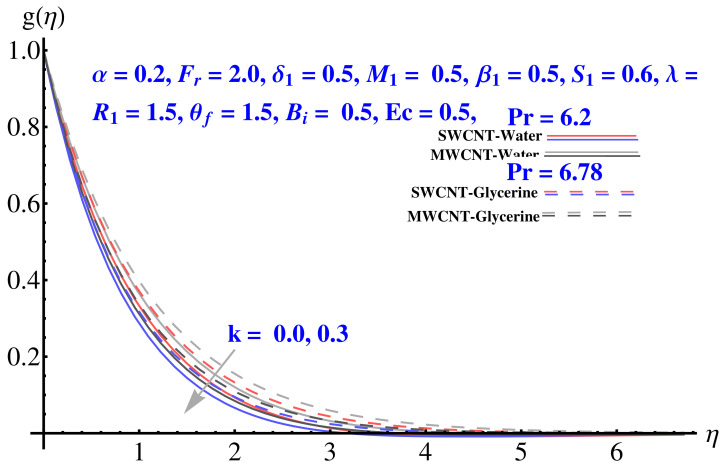
Impact of *k* on tangential velocity.

**Figure 11:**
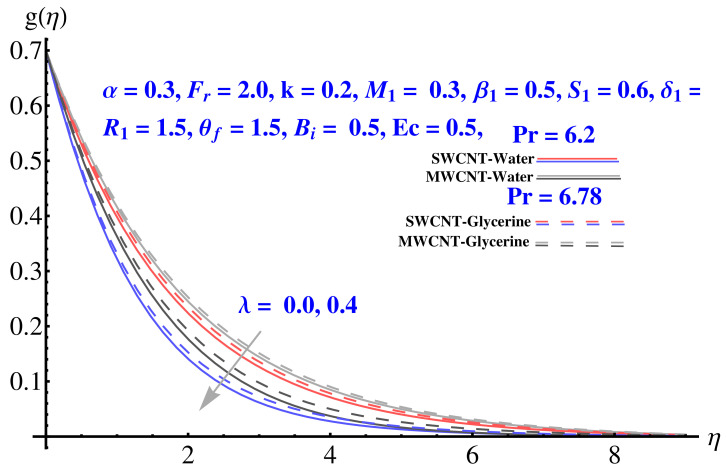
Impact of λ on tangential velocity.

Impact of various parameters on Tangential velocity profiles is given in Figure 7, Figure 8, Figure 9, Figure 10 and Figure 11. In particular, the impact of Casson factor on tangential velocity is given in Figure 7. A larger Casson factor results in decay of the transport rate. Subsequently, a shrinkage appeared in the corresponding boundary layer. Physically, the tensile stress appeared because of the elasticity yields a reduction in fluid movement. The stretching strength parameter results in the decline of the tangential velocity profile, as given in Figure 8. The impact of Forchheimer number, permeability parameter, and porosity parameter on the tangential velocity field is given in Figure 9, Figure 10 and Figure 11. In both cases, the larger values of corresponding parameters are found to be declining factors for the fluid velocity and the associated boundary layer shrinks up to a significant level. More resistance is offered to the fluid flow that causes disturbance in the smooth movement and, thereby, the velocity profile and associated boundary layer ends up with a reducing trend. The convective condition involved in the governing equations results in scattered diagrams of thermal profile at the boundary.

### 4.3. Temperature Field

**Figure 12:**
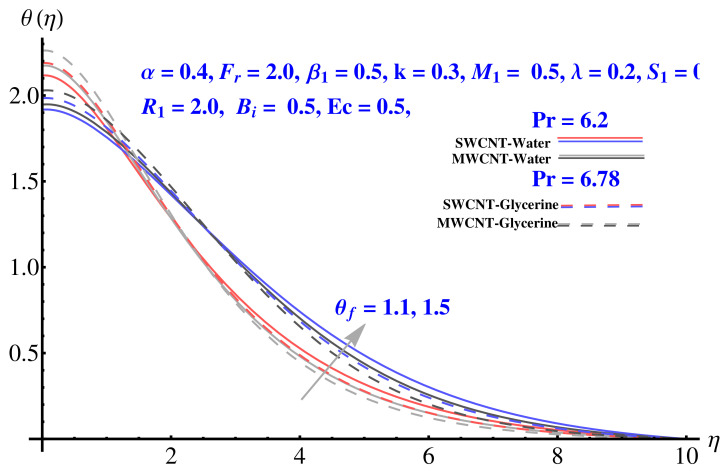
Impact of $\theta_{f}$ on temperature field.

**Figure 13:**
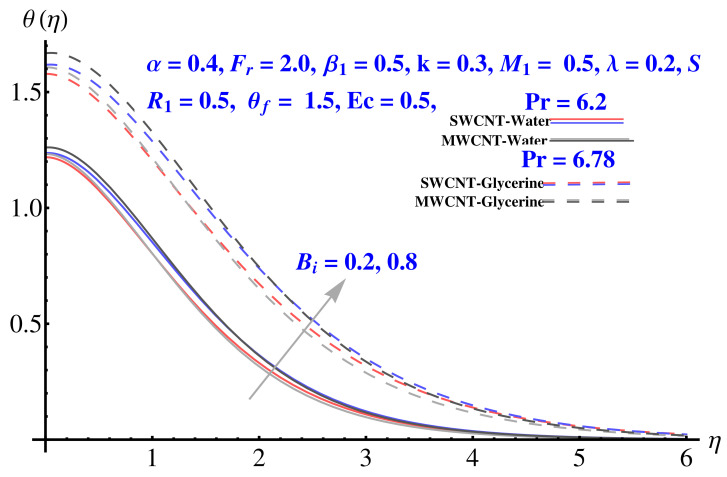
Impact of $B_{i}$ on temperature field.

**Figure 14:**
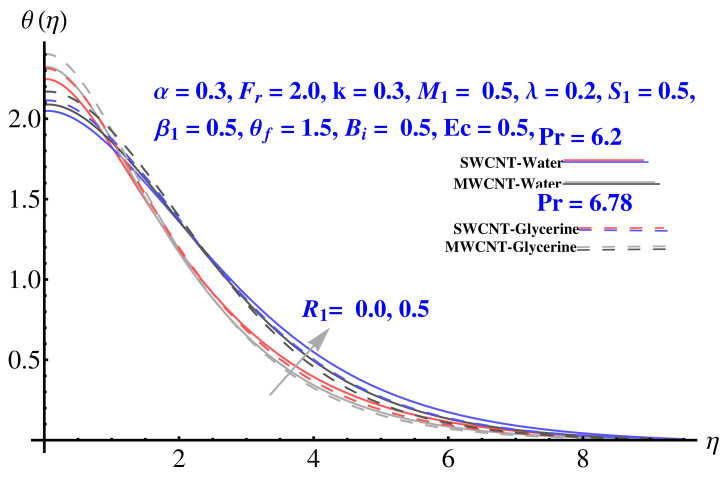
Impact of $R_{1}$ on temperature field.

The impact of various parameters on thermal profile is given in Figure 12, Figure 13 and Figure 14. The impact of $\theta_{f}$ on thermal profile is given in Figure 12. In both cases, the profile shows enhancement for elevated values of the corresponding parameter. A significant rise in thermal profile is noted for larger values of Biot number. The convective boundary results in enhancement of temperature facing the disk surface as compared to the ambient fluid. Physically, the trend of justified by the convective boundary. Similarly to Biot number, the enhanced radiation parameter results in more convenience in heat transfer rate and, therefore, the thermal state of the fluid enhances with larger values of radiation factor as given in Figure 14.

### 4.4. Contour and Density Graphs

**Figure 15:**
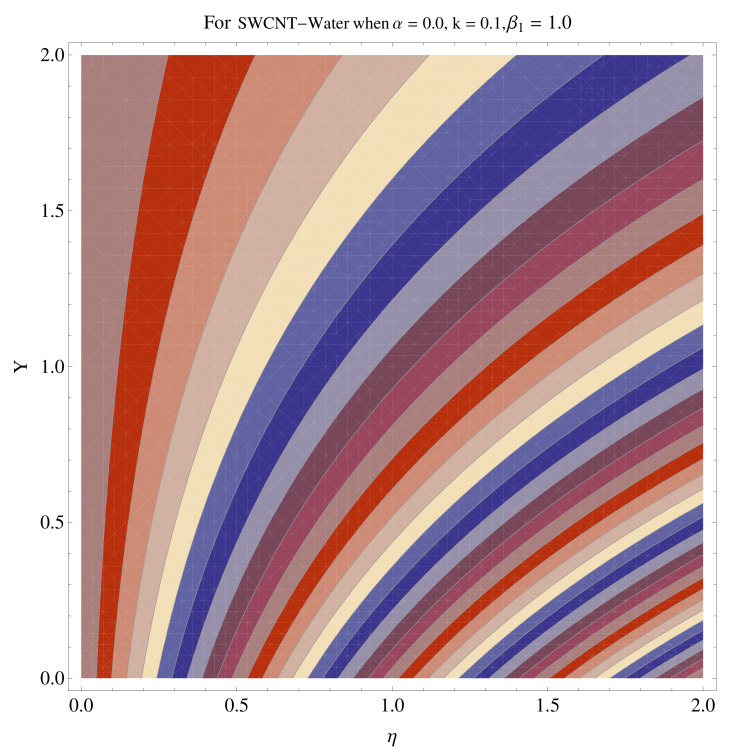
Contour graph at $\beta_{1}=1.0$ for SWCNT.

**Figure 16:**
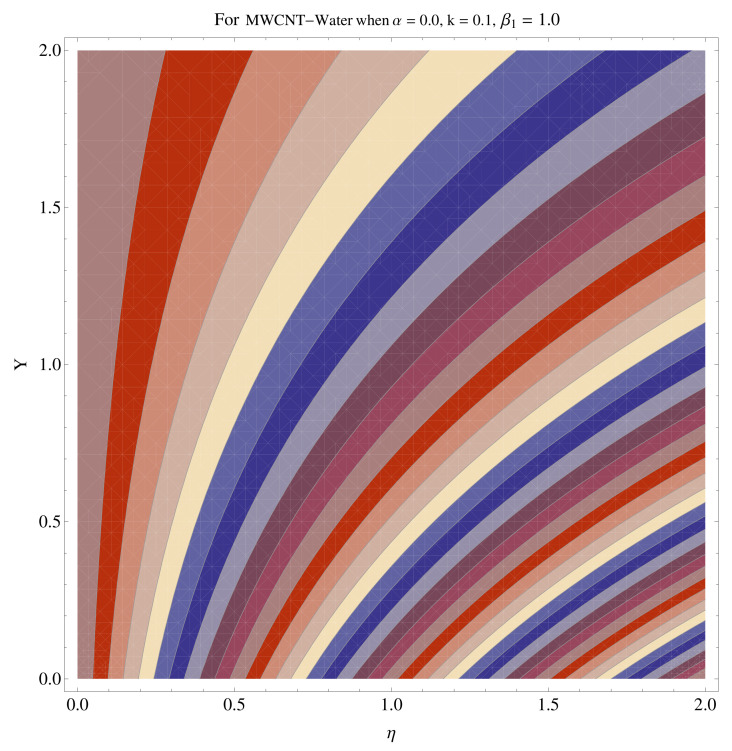
Contour graph at $\beta_{1}=1.0$ for MWCNT.

**Figure 17:**
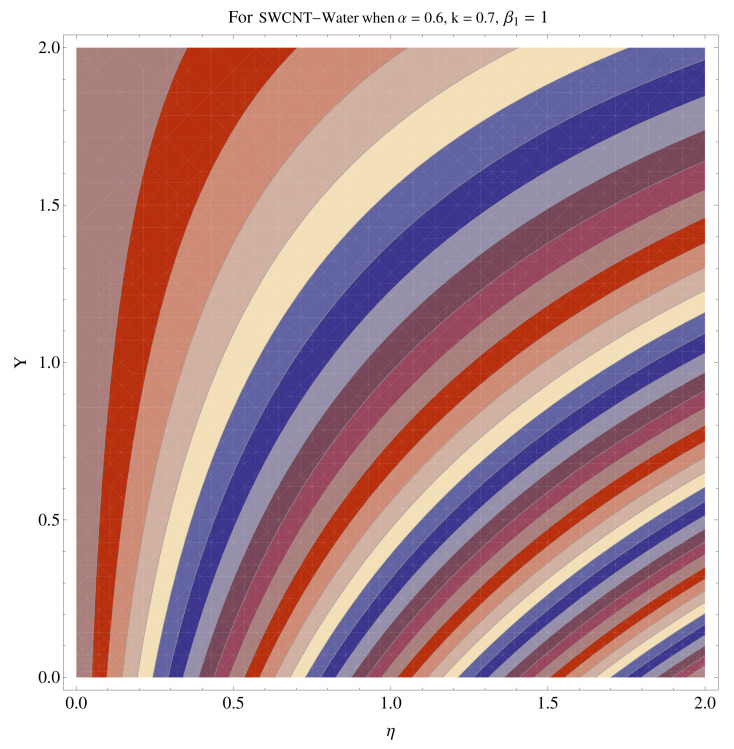
Contour graph at $\beta_{1}=1.0$ for SWCNT at larger *k*.

**Figure 18:**
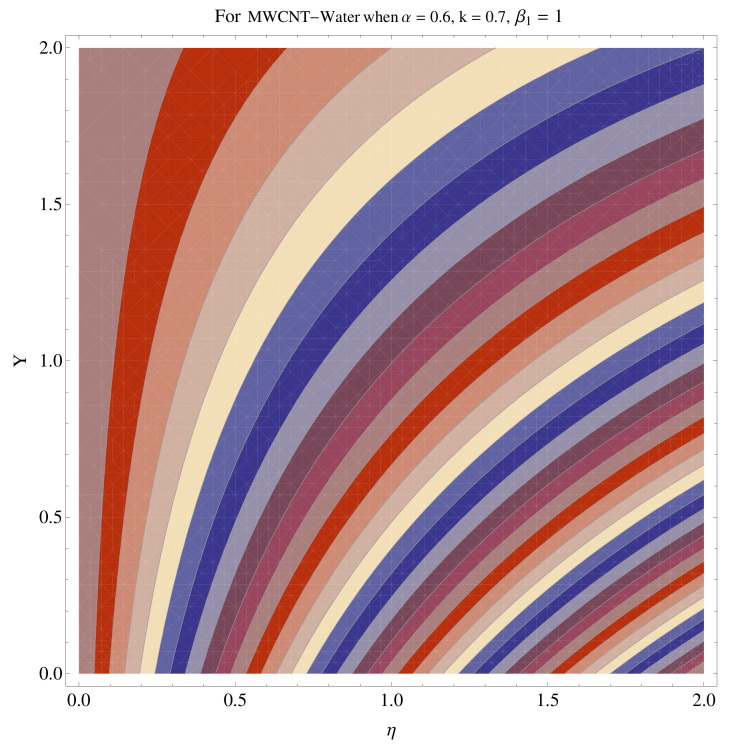
Contour graph at $\beta_{1}=1.0$ for MWCNT at larger *k*.

**Figure 19:**
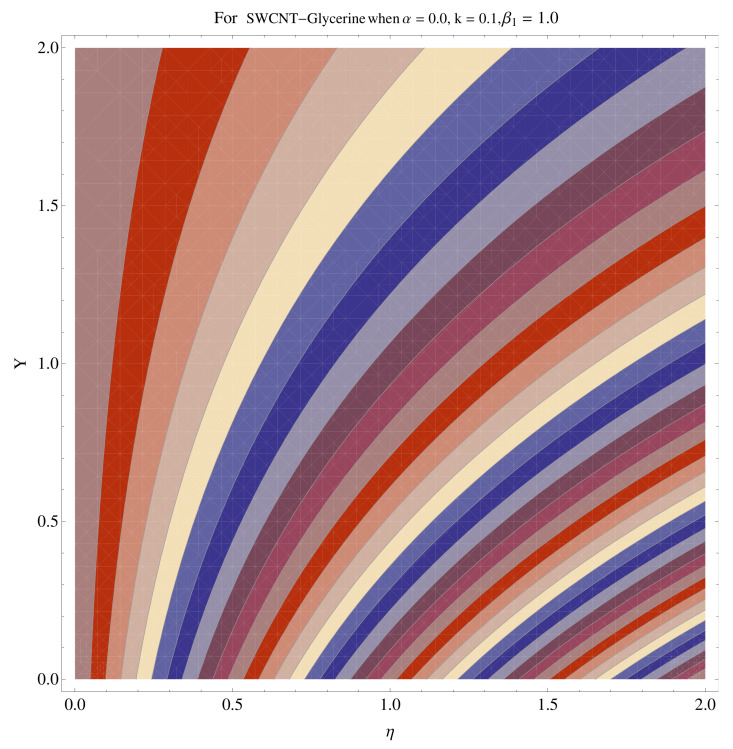
Contour graph at $\beta_{1}=1.0$ for SWCNT-Glycerine.

**Figure 20:**
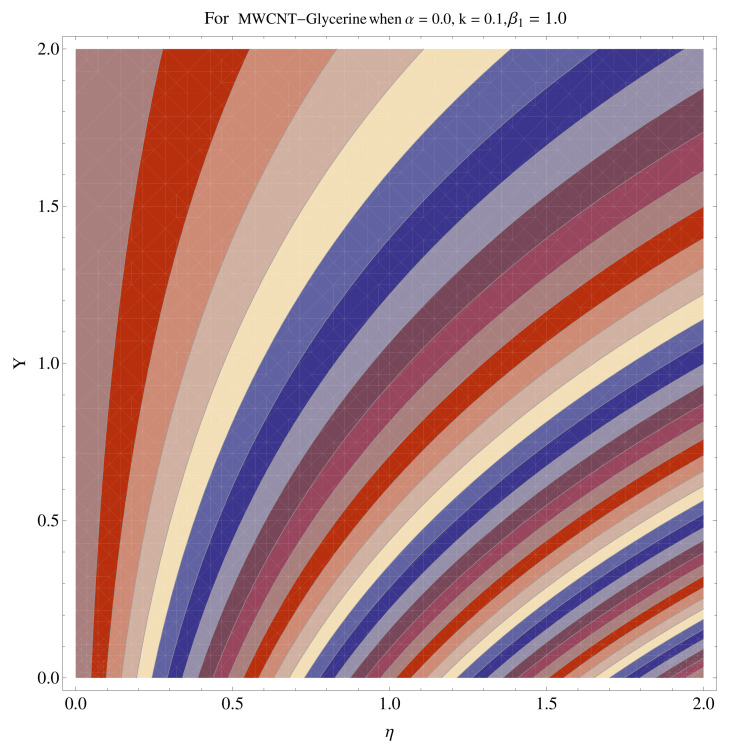
Contour graph at $\beta_{1}=1.0$ for MWCNT-Glycerine.

**Figure 21:**
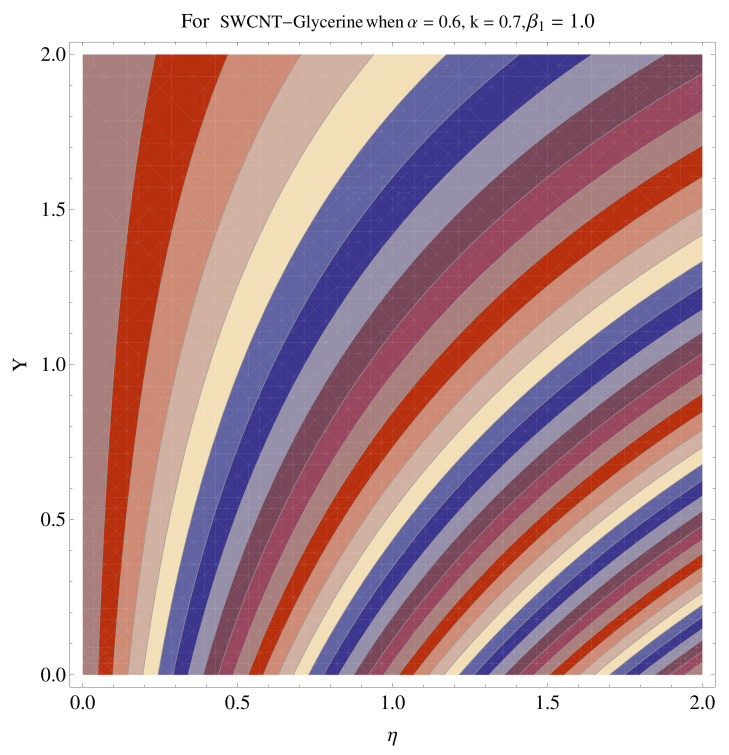
Contour graph at $\beta_{1}=1.0$ for SWCNT-Glycerine at larger *k*.

**Figure 22:**
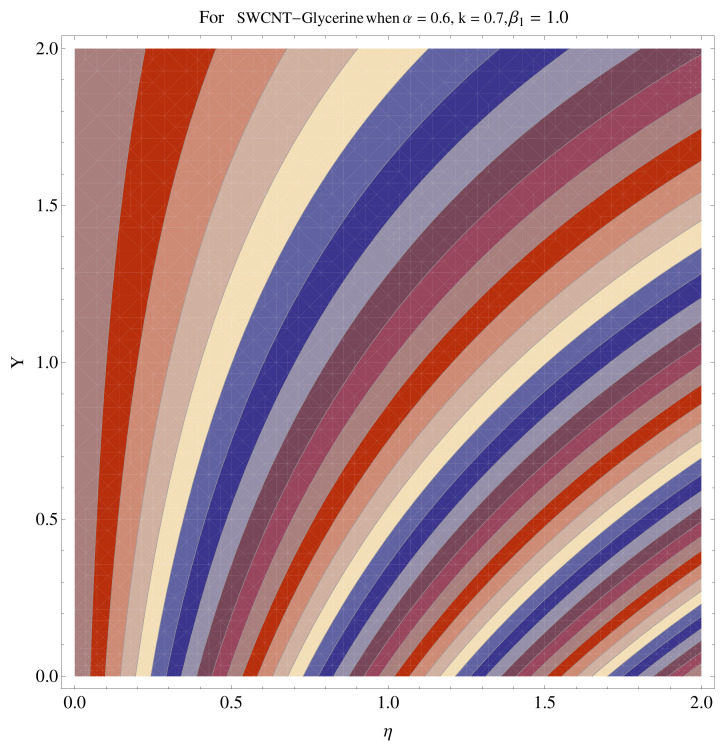
Contour graph at $\beta_{1}=1.0$ for MWCNT-Glycerine at larger *k*.

**Figure 23:**
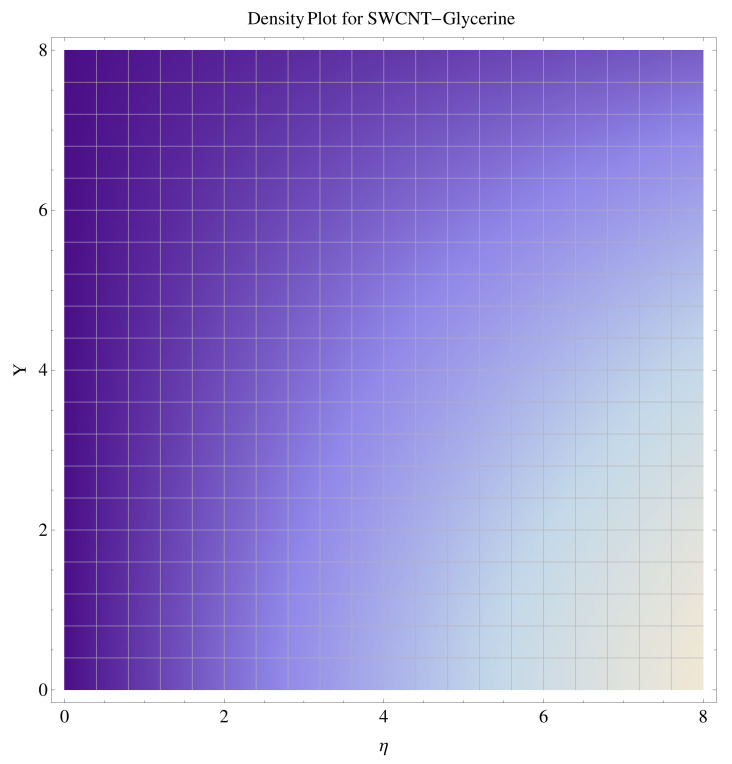
Density graph of single-walled nanotubes—Glycerine dilute.

**Figure 24:**
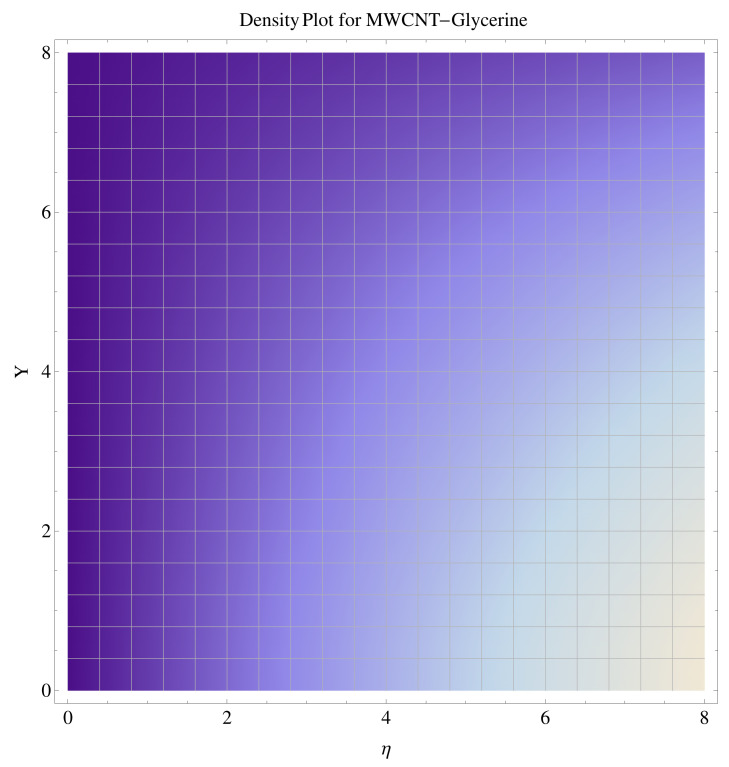
Density graph of multi-walled nanotubes—Glycerine dilute.

In Figure 15, Figure 16, Figure 17, Figure 18, Figure 19, Figure 20, Figure 21 and Figure 22, the contour graphs have been sketched for various values of Casson parameter and permeability parameters against the single- and multi-walled Carbon nanotubes—Water/Glycerine dilution. Results are prominent near the surface, as compared to away from the surface. Figure 23 and Figure 24 are the density graphs for both the SWCNT and MWCNT based Water/Glycerine nanofluid.

### 4.5. Skin Friction and Nusselt number

**Figure 25:**
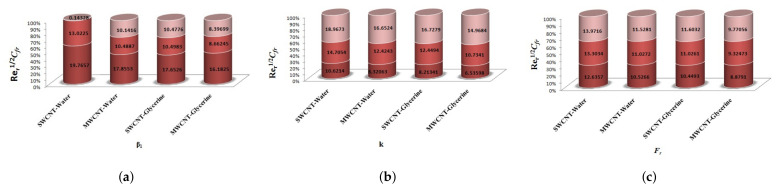
Impact of $\beta_{1},k,F_{r}$ on Skin-Friction.

**Figure 26:**
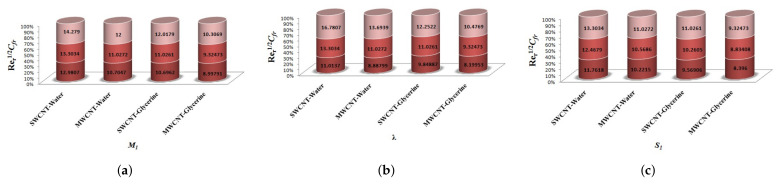
Impact of $M_{1},\lambda ,S_{1}$ on Skin-Friction.

**Figure 27:**
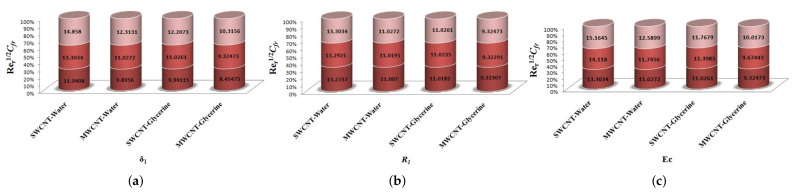
Impact of $\delta_{1},R_{1},Ec$ on Skin-Friction.

**Figure 28:**
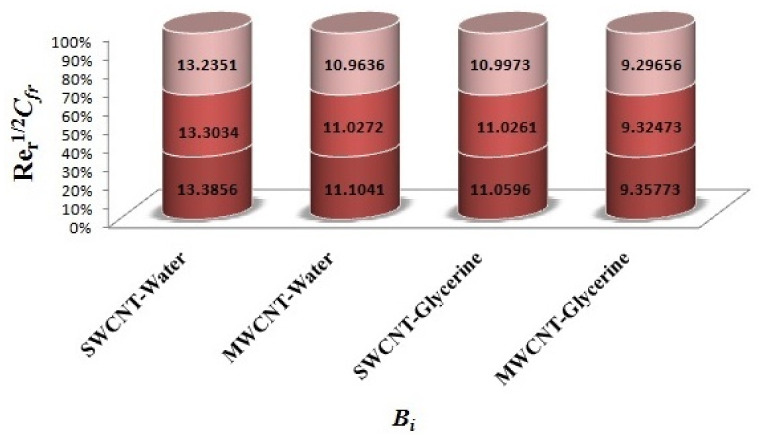
Impact of $B_{i}$ on Skin-friction.

**Figure 29:**
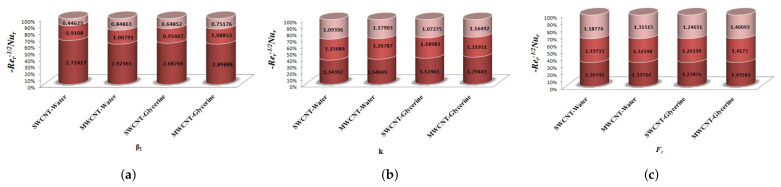
Impact of $\beta_{1},k,F_{r}$ on Nusselt number.

**Figure 30:**
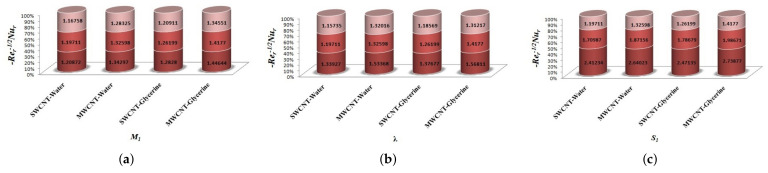
Impact of $M_{1},\lambda ,S_{1}$ on Nusselt number.

**Figure 31:**
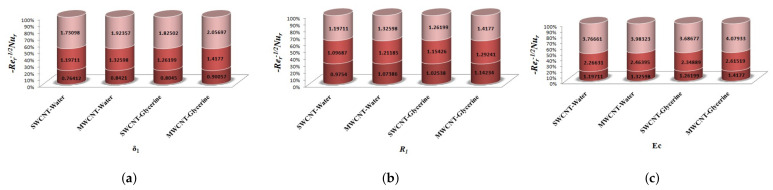
Impact of $\delta_{1},R_{1},Ec$ on Nusselt number.

**Figure 32:**
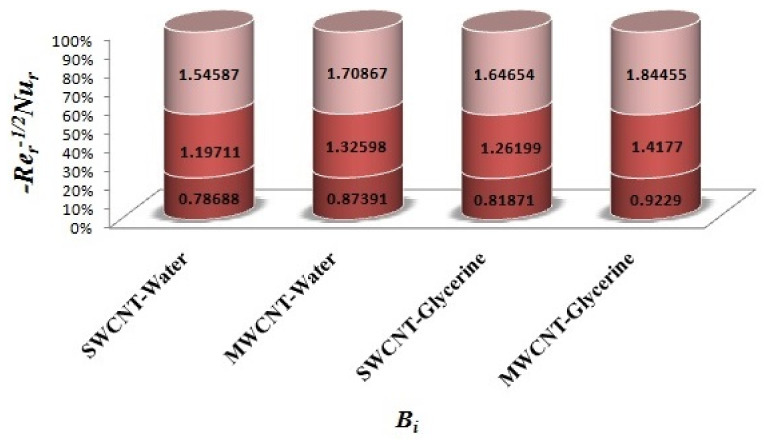
Impact of $B_{i}$ on Nusselt number.

The variation of Skin-friction and Nusselt number is given in graphical, as well as tabular date form in Figure 25, Figure 26, Figure 27, Figure 28, Figure 29, Figure 30, Figure 31 and Figure 32 and in Table 2 and Table 3. One can see an enhancement in Skin-friction for larger values of Forchheimer number. Similarly, the non-linear radiation parameter shows an increasing trend in skin-friction. However, the friction faces a decline for enhancement in Casson parameter. The Forchheimer number results in the decline of the nusselt number (heat flux) as compared to the skin-friction. Whereas, the non-linear radiation parameter significantly increases the heat flux rate. Similar to skin-friction, Casson parameter results in decline of heat flux.

## 5. Conclusions

The present investigation aims to reveal the significance of non-linear thermal radiation on Casson-water/glycerine MHD Darcy-Forchheimer fluid flow analysis subject to a rotating frame. The rate of heat transfer and skin-friction are analyzed. The graphical display of results gives the impact of various parameters involved in the flow model on the main profiles of momentum and energy. The numerical RK45 scheme is invoked to obtain the requisite solutions of the governing non-linear ordinary differential equations. Salient features are listed below:

- The enhanced Forcheimer number results in a decline in radial velocity;
- Casson parameter restricts the fluid velocity at larger values;
- The larger the porosity parameter, the larger the resistance offered by the medium to the fluid flow and the consequent result is the decline in velocity;
- The larger permeability parameter (K) results a drastic declination in both cases (SWCNT and MWCNT) when the values of K are increased;
- A larger Casson factor results in the shrinkage in the corresponding boundary layer of thermal field;
- Forchheimer number, permeability parameter and porosity parameter on tangential decrease the velocity field;
- The convective boundary results in enhancement of temperature facing the disk surface as compared to the ambient fluid;
- The contour graphs have been sketched for various values of Casson parameter and permeability parameter against the single- and multi-walled Carbon nanotubes—Water/Glycerine nanofluid.
- Skin-friction for larger values of Forchheimer number.
- The non-linear radiation parameter significantly increases the heat flux rate.

## Figures and Tables

**Table 1:** Thermophysical properties of fluid and nanoparticles (for reference see Sheikholeslami et al. [5]).

	Base Fluid					Nanoparticles	
**Properties**	**Water**	**Kerosene**	**Glycerin**	**Ethylene Glycol**	**Engine** **Oil**	**SWCNT**	**MWCNT**
$C_{p}$ (J/kg K)	4179	2090	2427	2430	1910	427	796
ρ (KG/m${}^{3}$)	997	783	1259.9	1115	884	2600	1600
*k* (W/mK)	0.613	0.145	0.286	0.253	0.144	6600	3000
$\beta \times 10^{-5}$	21		48	65	70	27	44
Pr	6.2	21	6.78	203.63	6450	−	−

**Table 2:** Skin friction for water and Glycerine $\theta_{f}=1.5$.

										Rer1/2Cfr			
$\beta_{1}$	*k*	$F_{r}$	$M_{1}$	λ	$S_{1}$	$\delta_{1}$	$R_{1}$	Ec	$B_{i}$	*Water*		*Glycerine*	
										*SWCNT*	*MWCNT*	*SWCNT*	*MWCNT*
0.1	0.2	2.5	0.5	0.2	0.6	0.7	2.0	0.3	0.5	19.7657	17.8553	17.6526	16.1825
1.0										13.0225	10.4887	10.4983	8.66245
4.5										0.14328	10.1416	10.4776	8.39699
0.5	0.0	2.5	0.5	0.2	0.6	0.7	2.0	0.3	0.5	10.6214	8.32063	8.21341	6.53598
	0.3									14.7054	12.4243	12.4494	10.7341
	0.6									18.9673	16.6524	16.7279	14.9684
0.5	0.2	2.0	0.5	0.2	0.6	0.7	2.0	0.3	0.5	12.6357	10.5266	10.4493	8.87910
		2.5								13.3034	11.0272	11.0261	9.32473
		3.0								13.9716	11.5281	11.6032	9.77056
0.5	0.2	2.5	0.0	0.2	0.6	0.7	2.0	0.3	0.5	12.9807	10.7047	10.6962	8.99791
			0.5							13.3034	11.0272	11.0261	9.32473
			1.0							14.2790	12.0000	12.0179	10.3069
0.5	0.2	2.5	0.5	0.0	0.6	0.7	2.0	0.3	0.5	11.0137	8.88799	9.84887	8.19953
				0.2						13.3034	11.0272	11.0261	9.32473
				0.4						16.7807	13.6939	12.2522	10.4769
0.5	0.2	2.5	0.5	0.2	$0.0S_{1}$	0.7	2.0	0.3	0.5	11.7618	10.2215	9.56906	8.39600
					0.3					12.4679	10.5686	10.2605	8.83408
					0.6					13.3034	11.0272	11.0261	9.32473
0.5	0.2	2.5	0.5	0.2	0.6	0.6	2.0	0.3	0.5	11.9408	9.8956	9.99115	8.45475
						0.7				13.3034	11.0272	11.0261	9.32473
						0.8				14.858	12.3131	12.2073	10.3156
0.5	0.2	2.5	0.5	0.2	0.6	0.7	1.0	0.3	0.5	13.2737	11.007	11.0182	9.31907
							1.5			13.2921	11.0195	11.0235	9.32291
							2.0			13.3034	11.0272	11.0261	9.32473
0.5	0.2	2.5	0.5	0.2	0.6	0.7	2.0	0.3	0.5	13.3034	11.0272	11.0261	9.32473
								0.6		14.1180	11.7456	11.3985	9.67443
								0.9		15.1645	12.5899	11.7679	10.0173
0.5	0.2	2.5	0.5	0.2	0.6	0.7	2.0	0.3	0.1	13.3856	11.1041	11.0596	9.35773
									0.3	13.3034	11.0272	11.0261	9.32473
									0.6	13.2351	10.9636	10.9973	9.29656

**Table 3:** Nusselt number for water and Glycerine $\theta_{f}=1.5$.

										−Rer−1/2Nur			
$\beta_{1}$	*k*	$F_{r}$	$M_{1}$	λ	$S_{1}$	$\delta_{1}$	$R_{1}$	Ec	$B_{i}$	*Water*		*Glycerine*	
										*SWCNT*	*MWCNT*	*SWCNT*	*MWCNT*
0.1	0.2	2.5	0.5	0.2	0.6	0.7	2.0	0.3	0.5	2.72417	2.92365	2.68766	2.89888
1.0										0.91080	1.00791	0.95407	1.08853
4.5										0.44625	0.84863	0.64852	0.75176
0.5	0.0	2.5	0.5	0.2	0.6	0.7	2.0	0.3	0.5	1.34392	1.54669	1.52461	1.79443
	0.3									1.15684	1.26787	1.18983	1.31951
	0.6									1.09396	1.17903	1.07275	1.16492
0.5	0.5	2.0	0.5	0.2	0.6	0.7	2.0	0.3	0.5	1.20741	1.33764	1.27875	1.43563
		2.5								1.19711	1.32598	1.26199	1.41770
		3.0								1.18774	1.31515	1.24651	1.40093
0.5	0.2	2.5	0.0	0.2	0.6	0.7	2.0	0.3	0.5	1.20872	1.34297	1.28280	1.44644
			0.5							1.19711	1.32598	1.26199	1.41770
			0.6							1.16758	1.28325	1.20911	1.34551
0.5	0.2	2.5	0.5	0.0	0.6	0.7	2.0	0.3	0.5	1.33927	1.53368	1.37677	1.56811
				0.2						1.19711	1.32598	1.26199	1.41770
				0.4						1.15735	1.32016	1.18569	1.31217
0.5	0.2	2.5	0.5	0.2	0.0	0.7	2.0	0.3	0.5	2.41234	2.64023	2.47135	2.73877
					0.3					1.70987	1.87156	1.78679	1.98671
					0.6					1.19711	1.32598	1.26199	1.41770
0.5	0.2	2.5	0.5	0.2	0.6	$\delta_{1}0.6$	2.0	0.3	0.5	0.76412	0.84210	0.80450	0.90057
						0.7				1.19711	1.32598	1.26199	1.41770
						0.8				1.73098	1.92357	1.82502	2.05697
0.5	0.2	2.5	0.5	0.2	0.6	0.7	1.0	0.3	0.5	0.97540	1.07386	1.02538	1.14234
							1.5			1.09687	1.21185	1.15426	1.29241
							2.0			1.19711	1.32598	1.26199	1.41770
0.5	0.2	2.5	0.5	0.2	0.6	0.7	2.0	0.3	0.5	1.19711	1.32598	1.26199	1.41770
								0.6		2.26631	2.46395	2.34889	2.61519
								0.9		3.76661	3.98323	3.68677	4.07933
0.5	0.2	2.5	0.5	0.2	0.6	0.7	2.0	0.3	0.1	0.78688	0.87391	0.81871	0.92290
									0.3	1.19711	1.32598	1.26199	1.41770
									0.6	1.54587	1.70867	1.64654	1.84455

## Nomenclature

MHD	Magnetohydrodynamic
ODEs	Ordinary Differential Equations
PDEs	Partial Differential Equation
CNTs	Carbon Nanotubes
$\rho_{nf}$	density of given nanofluid
$\rho_{CNTs}$	density of carbon nanotubes
u,v,w	Velocity components/m·s${}^{-1}$
*P*	Pressure /Pa
$\mu_{f}$	dynamic viscosity of considered base fluid /kgm${}^{-1}$ s${}^{-1}$
$\mu_{nf}$	dynamic viscosity of the given nanofluid /kgm${}^{-1}$ s${}^{-1}$
*T*	Local temperature/K
$T_{\infty }$	Ambient temperature/K
f,g	Nondimensional velocity components
$g_{1}$	Gravitational force
Gr	Grashof number
*K*	Permeability parameter
Fr	Forchheimer number (inertia)
$\beta_{1}$	Casson parameter
ϕ	Volume fraction (solid)
Ω	Angular Velocity (angular)
$C_{f}$	Skin-friction (wall drag force)
$Nu_{x}$	Local Nusselt number
$Re_{x}$	Local Reynolds number
$M_{1}$	magnetic number
Pr	Prandtl number
Ec	Eckert parameter
λ	Ratio of Grashof and Reynolds square
$S=\frac{W}{\sqrt{2\Omega v_{f}}}$	Suction parameter
